# Clinical features and prognostic factors in 71 eyes over 20 years from patients with Coats’ disease in Korea

**DOI:** 10.1038/s41598-021-85739-9

**Published:** 2021-03-17

**Authors:** Hyun Goo Kang, Jung Dong Kim, Eun Young Choi, Suk Ho Byeon, Sung Soo Kim, Hyoung Jun Koh, Min Kim

**Affiliations:** 1 Department of Ophthalmology, Institute of Vision Research, Gangnam Severance Hospital, Yonsei University College of Medicine, Eonjuro 211, Gangnam-gu, Seoul, 06273 Republic of Korea; 2 Translational Genome Informatics Laboratory, Department of Biomedical Systems Informatics, Yonsei University College of Medicine, Yonsei-ro 50-1, Seodaemun-gu, Seoul, Republic of Korea; 3 Department of Ophthalmology, Institute of Vision Research, Severance Eye Hospital, Yonsei University College of Medicine, Yonsei-ro 50-1, Seodaemun-gu, Seoul, Republic of Korea

**Keywords:** Diseases, Eye diseases, Paediatrics, Paediatric research

## Abstract

This retrospective study assesses the clinical features, treatment strategies, and long-term outcomes of patients with Coats’ disease in Korea. Multimodal imaging and medical records of consecutive patients treated between July 2000 and April 2020 at two tertiary centers were evaluated based on onset age (adult vs. childhood [< 18 years]). Factors associated with final visual acuity (VA) and risk of treatment failure were assessed. A total of 71 eyes of 67 patients were included, with subgrouping by onset age showing 45% childhood and 55% adult cases. Overall, Stage 2 disease was most common at presentation (76%), though childhood cases had more Stage 3b (22% vs. 3%, *P* = 0.02) and greater clock hours of retinal telangiectasia (7 vs. 5, *P* = 0.005). First-line treatment included laser (25%), combined laser/anti-VEGF (23%), cryotherapy (20%), surgery (16%), and anti-VEGF only (9%). Cryotherapy was associated with a higher risk for secondary interventions (OR 11.8, *P* < 0.001), required in 56% overall. Despite a 3-line VA decrease in 34% overall, adult cases had superior final VA (*P* = 0.037). Multivariable regression showed that the number of anti-VEGF injections performed during the initial treatment period was associated with a 9.4 letter improvement in vision (*P* = 0.041). We observed a higher proportion of adult-onset Coats’ disease than previously reported in other non-Asian populations. An aggressive treatment with the addition of anti-VEGF may yield the most favorable long-term visual outcomes.

## Introduction

Coats’ disease, a congenital idiopathic retinal vasculopathy first described in 1908, is characterized by retinal vascular telangiectasias and exudative retinopathy. While this disorder most commonly affects young men^1^, a less severe form with a slower rate of progression has been described in older patients and has been termed adult-onset Coats’ disease^2^. Typical clinical features of this disorder include subretinal and intraretinal exudates and exudative retinal detachment, with irregular, lightbulb-type vascular telangiectasias in the periphery, usually observed best on fluorescein angiography^1,3^. At times, this disorder can simulate retinoblastoma by producing localized exudation^4^.

The long-term visual prognosis for Coats’ disease is poor, especially in eyes with exudates involving the macula, and its recurrent nature may require life-long surveillance^5^. Treatment has become more sophisticated over time, transitioning from cryotherapy to laser ablation, leading to improved anatomical and functional outcomes; more recently, adjunctive therapy such as anti-vascular endothelial growth factor (anti-VEGF) injections have also become available for use^6^.

Unfortunately, there are few studies of Coats’ disease based mainly in Asian populations, despite potentially substantial geographic differences in the incidence, characteristics, and outcomes in patients with this disease. Therefore, this study provides a comprehensive overview of our 20-year experience managing Coats’ disease in an Asian population, assessing clinical factors, treatment strategies, and long-term outcomes based on age at disease presentation.

## Results

A total of 71 eyes from 67 ethnically Korean patients were included, with 30 patients (32 eyes, 45%) having childhood-onset disease and 37 patients (39 eyes, 55%) having adult-onset disease. The mean age at presentation was 8.6 years (median 8.7 years, range 0.2–17.8) in the childhood-onset subgroup and 39.8 years (median 39.8 years, range 18.3–69.0) in the adult-onset subgroup. Patient baseline characteristics, presenting symptoms/signs, initial visual acuity, and onset disease staging are summarized in Table 1. No patients with bilateral presentations had coexisting eye diseases or medical illnesses.

**Table 1:** Baseline characteristics of patients with Coats’ disease.

Baseline characteristic	Overall (n = 67 patients)	Subgroup analysis based on age at disease onset		
		Childhood (n = 30 patients)	Adult (n = 37 patients)	*P*-value^a^
No. eyes (%)	71 (100)	32 (45)	39 (55)	0.24
Age, years, mean ± SD	25.8 ± 19.9	8.6 ± 4.7	39.8 ± 16.1	< 0.001*
Female sex, n (%)	17 (25)	7 (23)	10 (27)	0.78
Right eye, n (%)	39 (55)	15 (47)	24 (62)	0.24
Bilateral, n (%)	6 (9)	4 (13)	2 (5)	0.40
**Presenting symptoms/signs, n (%)**				
Decreased vision	29 (43)	14 (47)	15 (41)	0.63
Floaters	11 (16)	2 (7)	9 (24)	0.10
Visual blurring	8 (12)	2 (7)	6 (16)	0.28
Strabismus	5 (8)	5 (17)	0 (0)	0.015*
Conjunctival injection or pain	1 (2)	0 (0)	1 (3)	> 0.99
Metamorphopsia	1 (1)	0 (0)	1 (3)	> 0.99
Leukocoria	1 (1)	1 (3)	0 (0)	> 0.99
Routine examination	8 (12)	4 (13)	4 (11)	> 0.99
Initial vision, mean logMAR ± SD	0.91 ± 0.86	1.09 ± 1.02	0.80 ± 0.73	0.24
Mean Snellen equivalent	20/162	20/245	20/126	–
**Disease classification**^13^, **n (%)**				
Stage 2a	28 (39)	12 (38)	16 (41)	0.81
Stage 2b	26 (37)	10 (31)	16 (41)	0.46
Stage 3a1	7 (10)	2 (6)	5 (13)	0.45
Stage 3a2	2 (3)	1 (3)	1 (3)	> 0.99
Stage 3b	8 (11)	7 (22)	1 (3)	0.019*

*LogMAR *logarithm of minimum angle of resolution.

*P-values of < 0.05 are considered statistically significant.

^a^The chi-squared test and Student’s t-test.

The most common presenting sign or symptom in both groups was decreased visual acuity (overall 29 cases, 43%). Coats’ disease was discovered during routine examinations in eight patients. Visual acuities at presentation were similar in both groups, ranging from 20/20 to no light perception.

### Disease classification and vitreoretinal findings

Using the Shields classification system^7^, the disease staging at presentation was generally similar for both groups (Table 1), though more severe Stage 3b disease was observed significantly more frequently in the childhood-onset group (22%) than in the adult-onset group (3%) (*P* = 0.019). No cases with Stage 4 or 5 disease were observed in either group at presentation.

A summary of vitreoretinal findings at presentation can be found in Table 2. While patients in the childhood-onset group had a significantly higher proportion of ring exudation (*P* = 0.041) and significantly greater clock hours of retinal telangiectasias (*P* = 0.005) at presentation, retinal haemorrhages were more commonly observed in the adult-onset group (*P* = 0.030). CME was the most common abnormality identified on OCT in both groups (40%), with no significant differences in other OCT findings between groups.

**Table 2:** Vitreoretinal findings at presentation in patients with Coats’ disease.

Vitreoretinal finding	Overall (n = 71 eyes)	Subgroup analysis based on age at disease onset		
		Childhood (n = 32 eyes)	Adult (n = 39 eyes)	*P*-value^a^
**Location of main exudation, n (%)**				
Temporal quadrant	42 (59)	18 (56)	24 (62)	0.81
Macular area	38 (54)	19 (59)	19 (49)	0.47
Inferior quadrant	16 (23)	8 (25)	8 (21)	0.78
Superior quadrant	13 (18)	3 (9)	10 (26)	0.12
Nasal quadrant	7 (10)	3 (9)	4 (10)	> 0.99
Diffuse ring exudation	7 (10)	6 (19)	1 (3)	0.041*
**Vascular abnormalities**				
Retinal telangiectasia, clock hours, mean ± SD	5.7 ± 3.2	6.9 ± 3.5	4.7 ± 2.6	0.005*
Vitreous haemorrhage, n (%)	3 (4.2)	1 (3)	2 (5)	> 0.99
Retinal haemorrhage, n (%)	38 (54)	12 (39)	26 (67)	0.030*
Vitreoretinal fibrosis, n (%)	16 (23)	10 (32)	6 (16)	0.15
Retinal detachment, clock hours, mean ± SD	2.0 ± 4.1	3.0 ± 5.2	1.3 ± 2.8	0.089
Presence of subretinal fibrosis, n (%)	5 (8)	3 (13)	2 (6)	0.64
**Features on OCT**				
Cystoid macular oedema, n (%)	28 (40)	10 (32)	18 (46)	0.33
CFT, μm, mean ± SD	412 ± 221	552 ± 274	283 ± 128	0.67
Foveal subretinal fluid, n (%)	18 (26)	10 (32)	8 (21)	0.41
Epiretinal membrane, n (%)	8 (12)	3 (10)	5 (14)	0.72
Vitreomacular traction syndrome, n (%)	2 (3)	1 (3)	1 (3)	> 0.99
Temporal macula dragging, n (%)	1 (2)	0 (0)	1 (3)	> 0.99
Foveal hypoplasia, n (%)	1 (2)	1 (3)	0 (0)	0.46

*CFT *central foveal thickness, *logMAR *logarithm of minimum angle of resolution, *OCT *optical coherence tomography.

*P-values of < 0.05 are considered statistically significant.

^a^The chi-squared test and Student’s t-test.

### Treatment

**Figure 1:**
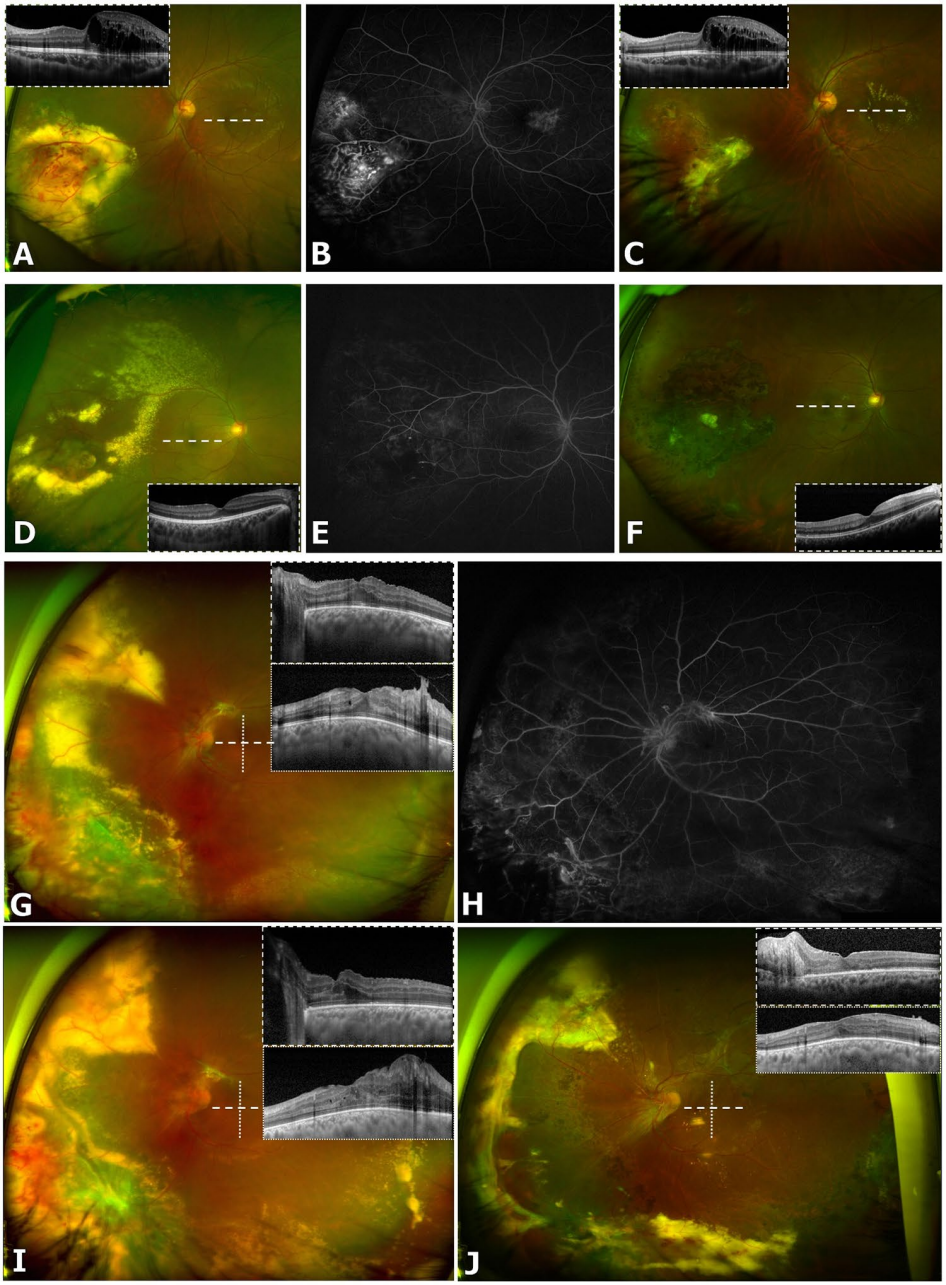
Ophthalmic imaging findings in representative cases of patients with Coats’ disease treated with a combination of laser ablation and anti-vascular endothelial growth factor (anti-VEGF) injections. Case 1 (**A**–**C**): A 14-year-old boy with no prior medical history presented with decreased vision in his left eye beginning one month prior to presentation. (**A**) Retinal telangiectasias with exudates involving two clock hours in the inferonasal quadrant, as well as retinal thickening in the temporal macula, were noted on ultra-widefield fundus photography and optical coherence tomography (OCT) imaging (inlet). (**B**) Fluorescein angiography showed multiple polyps and late leakage in the corresponding areas. (**C**) After a combination of six anti-VEGF injections and three laser sessions (including a macular focal laser), the peripheral lesions were stable on ultra-widefield fundus photography, though persistent exudative leakage was present in the macula on OCT. Case 2 (**D**–**F**): A man in his early thirties presented with floaters in his right eye and was diagnosed with Coats’ disease after review of ultra-widefield fundus photography (**D**), OCT (**D**, inlet), and fluorescein angiography (**E**). This patient underwent a combination of two laser sessions and three anti-VEGF injections. (**F**) Three years later, the patient was in a stable condition with no signs of disease reactivation or recurrence on ultra-widefield fundus photography and OCT (inlet). Case 3 (**G**–**J**): A 20-year-old was diagnosed with Coats’ disease, based on the peripheral lesions on ultra-widefield fundus photography (**G**) and OCT (inlet), as well as fluorescein angiography (**H**). A combination of two laser sessions and two anti-VEGF injections were performed in this patient. (**I**) Unfortunately, progression of both macular fibrosis with traction and peripheral exudation were observed, and the patient underwent vitrectomy for removal of vitreoretinal fibrotic membranes. (**J**) At 1-year post-vitrectomy, there were no signs of disease recurrence or reactivation, although vitreoretinal fibrosis was observed in the superior quadrant.

First-line treatment modalities are summarized in Table 3. While significantly more childhood-onset cases were initially treated with cryotherapy (*P* = 0.007), more adult-onset cases were initially treated using a combination of laser therapy and anti-VEGF injections (*P* = 0.004). Though not statistically significant, a greater number of eyes in the childhood-onset group presented at a more severe disease stage requiring initial surgical interventions (vitrectomy and/or scleral buckle) (*P* = 0.055). Initial treatment with anti-VEGF injections only (mean 2.2 injections, range 1–4) or observation only involved eyes with minimal exudation and relatively mild disease with little or no progression. There were no cases that underwent primary enucleations. Typical imaging findings from representative cases can be found in Fig. 1.

**Table 3:** Treatment and outcomes of patients with Coats’ disease.

Treatment/outcome	Overall (n = 67 patients)	Subgroup analysis based on age at disease onset		
		Childhood (n = 30 patients)	Adult (n = 37 patients)	*P*-value^a^
**First-line treatment strategy, n (%)**				
Laser only	18 (25)	7 (22)	11 (28)	0.59
Combined laser + anti-VEGF	16 (23)	2 (6)	14 (36)	0.004*
Cryotherapy	14 (20)	12 (38)	3 (8)	0.007*
Vitrectomy or scleral buckle	11 (16)	8 (25)	3 (8)	0.055
Anti-VEGF only	6 (9)	1 (3)	5 (13)	0.21
Observation	5 (7)	2 (6)	2 (8)	> 0.99
Secondary interventions required, n (%)	40 (56)	18 (56)	22 (56)	> 0.99
Laser	21 (30)	11 (34)	10 (26)	0.45
Surgery (vitrectomy, buckle)	18 (25)	8 (25)	10 (26)	> 0.99
Cryotherapy	13 (18)	7 (22)	6 (15)	0.55
Anti-VEGF	11 (16)	3 (9)	8 (21)	0.32
Corticosteroid injection	5 (7)	1 (3)	4 (10)	0.37
**Complications**				
Progression of vitreoretinal fibrosis	33 (52)	18 (67)	15 (41)	0.047*
Cataract surgery	9 (13)	4 (13)	5 (13)	> 0.99
Persistent CME	6 (9)	3 (9)	3 (8)	> 0.99
Phthisis	3 (4)	3 (9)	0 (0)	0.087
Disciform scarring or macular atrophy	3 (4)	1 (3)	2 (5)	> 0.99
Corneal opacity	3 (4)	1 (3)	2 (5)	> 0.99
Secondary ERM surgery	3 (4)	0 (0)	3 (4)	0.25
Enucleation	2 (3)	1 (3)	1 (3)	> 0.99
Glaucoma surgery	1 (1)	1 (3)	0 (0)	0.45
Follow-up duration, months, mean ± SD	75.2 ± 61.7	104.1 ± 70.8	51.7 ± 41.0	< 0.001*
Final vision, mean logMAR ± SD	1.05 ± 0.92	1.36 ± 1.09	0.85 ± 0.75	0.037*
Mean Snellen equivalent (range)	20/223 (NLP-20/20)	20/458 (NLP-20/20)	20/141 (NLP-20/20)	
Greater than 3-line decrease, n (%)	22 (34)	11 (39)	11 (31)	0.60

*CME *cystoid macular oedema, *ERM *epiretinal membrane, *TRD *tractional retinal detachment, *VEGF *vascular endothelial growth factor.

*P-values of < 0.05 are considered statistically significant.

^a^The chi-squared test and Student’s t-test.

Eyes initially treated with laser therapy only (18 eyes, 25%) received an average of 2.1 sessions (range 1–6) compared to those who underwent a combination of initial treatments (16 eyes, 23%) involving laser therapy (mean 1.6 sessions, range 1–3) and anti-VEGF injections (mean 2.3 injections, range 1–6). No significant differences were observed between groups in terms of the number of laser sessions (laser only: *P* = 0.99, laser in combination: *P* = 0.26) or anti-VEGF injections (*P* = 0.23).

Additionally, adjuvant intravitreal or sub-Tenon corticosteroid injections (mean 2 injections, range 1–4; *P* = 0.17 between groups) were performed as a supplemental first-line treatment in six eyes (9%) overall, with three in the childhood-onset (9%) and three in the adult-onset (8%) group.

**Figure 2:**
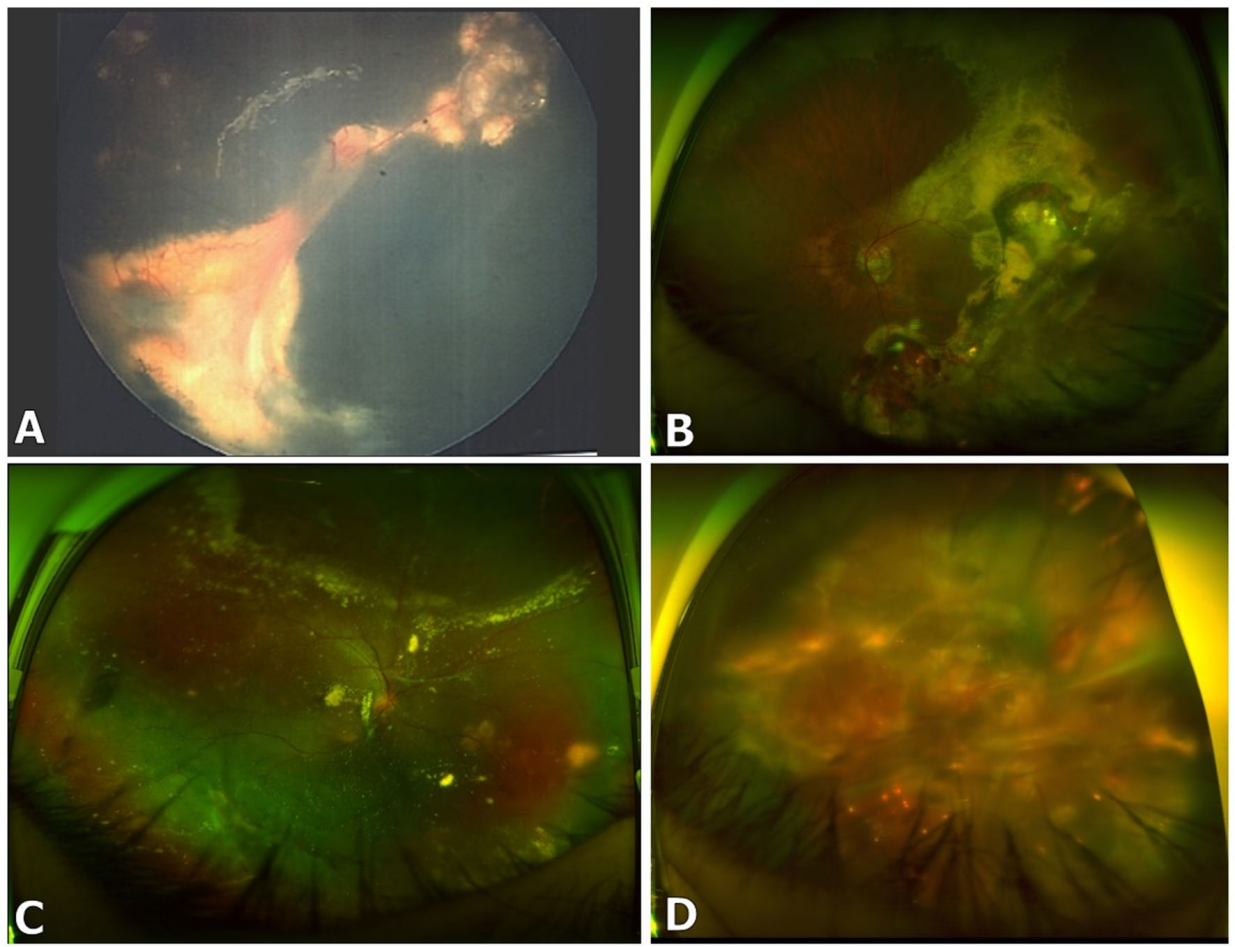
Tractional retinal detachments in Coats’ disease. Patients may develop tractional detachments during long-term follow-up after treatment with cryotherapy (**A**), laser (**B**), or laser combined with anti-VEGF injections (**C**,**D**).

Secondary interventions or retreatments were required in 40 eyes overall (56%, *P* > 0.99 between groups), with the most common cause being the occurrence or progression of tractional retinal detachments in 19 eyes (*P* > 0.79 between groups) (Fig. 2). The most common secondary treatment modality was laser therapy (30%, mean 1.4 sessions, range 1–3) followed by surgery (25%). Additional anti-VEGF injections (mean 3.3 injections, range 1–10) and/or corticosteroid injections (mean 2.8 injections, range 1–5) were also administered. Overall, no significant differences were observed between groups in the types of treatment modalities or the number of sessions/injections (all *P* > 0.05) that were required during secondary interventions.

### Complications and visual acuity

Patients were followed for a mean of 75.2 months (median 63 months, range 1–231), with longer follow-up for childhood-onset patients. Progression of vitreoretinal fibrosis, the most common complication, was observed significantly more frequently in childhood-onset eyes (*P* = 0.047). There were no other significant differences in complication rates between groups, even in terms of severe ocular comorbidities (Table 3).

The final mean visual acuity was 20/233 in the overall group (range 20/20 to no light perception) and was more favourable in the adult-onset group (*P* = 0.037). Over a third of all eyes (34%) experienced a greater than 3-line decrease in vision compared with baseline.

### Potential benefits of supplemental anti-VEGF injections

An additional subgroup analysis was performed to compare eyes treated using laser only (n = 18) to eyes treated using a combination of laser therapy and anti-VEGF injections (n = 16) in order to assess for potential benefits of anti-VEGF injections. There were no significant differences in the baseline demographics (age, sex), follow-up durations, retinal findings, or disease staging between these two subgroups (all *P* > 0.05). The rates of secondary interventions were also similar in these treatment groups (laser only vs. combined, 50% vs 69%, *P* = 0.32), and there were no statistical differences in the incidence of complications or in visual outcomes in these two groups (all *P* > 0.05). However, while not statistically significant, we can see a trend for superior final visual acuity in eyes treated with combination of laser and anti-VEGF (20/472 vs 20/99, *P* = 0.066).

### Factors associated with visual outcomes

Univariable linear regression revealed that the following factors were significantly associated with a more favourable final visual acuity: better visual acuity (β = 0.632, 95% confidence interval [CI] 0.384–0.879, *P* < 0.001), older age (β = − 0.013, − 0.024 to − 0.001, *P* = 0.032), less clock hours of retinal telangiectasia (β = 0.117, 0.046–0.189, *P* = 0.002), less clock hours of exudative retinal detachment (β = 0.091, 0.020–0.161, *P* = 0.013), lower central foveal thickness (β = 0.001, < 0.001 to 0.002, *P* = 0.033) at presentation, and more anti-VEGF injections during first-line treatment (β = 0.188, − 0.368 to − 0.009, *P* = 0.041). After controlling for age, multivariable linear regression using the stepwise method showed that only the number of anti-VEGF injections during initial primary treatment (β = − 0.188, 95% CI − 0.368 to − 0.009, *P* = 0.041), whether in combination with laser or anti-VEGF alone, was significantly associated with final visual acuity. Accordingly, with regards to the study population at our centres, we found that additional anti-VEGF injections performed during initial treatment period was associated with an improved final visual acuity by approximately 9.4 letters.

### Factors associated with secondary interventions

Both univariable and multivariable logistic regression analyses of factors associated with a need for secondary interventions can be found in Table 4. Using multivariable regression analysis, only the use of cryotherapy during initial treatment was significantly associated with a need for secondary treatment (odds ratio 11.774, *P* < 0.001).

**Table 4:** Factors associated with a need for secondary interventions in patients with Coats’ disease.

Factor	Univariable analysis		Multivariable analysis	
	OR (95% CI)	*P*-value^a^	OR (95% CI)	*P*-value^b^
Initial laser treatment	4.421 (1.354–14.436)	0.014*	3.916 (0.961–15.962)	0.057
Initial cryotherapy	12.133 (3.759–39.168)	< 0.001*	11.774 (2.991–46.340)	< 0.001*
Telangiectasia, clock hours	1.270 (1.020–1.581)	0.032*	1.003 (0.756–1.331)	0.98
Presence of diffuse ring exudation	0.100 (0.011–0.941)	0.044*	1.218 (0.066–22.424)	0.90

*CI *confidence interval, *OR *odds ratio.

* *P*-values of < 0.05 are considered statistically significant.

^a^Univariable logistic regression analysis.

^b^Multivariable logistic regression analysis.

## Discussion

In this study, we conducted a multicentre, retrospective review of medical records to investigate the long-term outcomes of patients with Coats’ disease managed using various treatment modalities over 20 years in Korea. In 71 eyes from 67 patients, we observed an overall earlier disease staging at presentation than previously reported in non-Asian populations, with a higher proportion of adult-onset disease and comparatively less severe vitreoretinal findings. Despite increasing usage of anti-VEGF injections and laser treatments as first-line therapeutic approaches, over half of cases (56%) required secondary interventions. We found that initial cryotherapy was significantly associated with disease reactivation and tractional retinal detachments requiring secondary interventions. Visual outcomes were more favourable in the adult-onset group, and adjunctive anti-VEGF injections were associated with improved final visual acuity.

In our study, the vast majority of cases presented with Stage 2 disease (76%), followed by Stage 3 disease (24%), with no patients presenting with Stage 4 or 5 disease. This finding contrasts with an American population-based study by Shields et al.^8^ that identified Stage 2 disease in 21% of patients, Stage 3 in 68% of patients, and Stages 4/5 in 7% of patients. Another Saudi Arabian population-based study by Al-Qahtani et al.^9^ described Stage 2 in 31%, Stage 3 in 56%, and Stages 4/5 in 12% of patients. Finally, a British population-based study by Morris et al.^10^ showed Stage 2 in 62%, Stage 3 in 34%, and Stages 4/5 in 7% of patients. Our differing results suggest that Coats’ disease is more likely to be detected at an earlier stage in Korea. Multiple factors may contribute to this earlier detection, including ease of access to primary care services in the universal healthcare system, a higher population density resulting in shorter distances to medical centres, and the mandatory vision testing that is performed when Korean children begin preschool and annually during general medical screenings^11^.

We observed a much higher proportion of adult-onset cases compared to other related studies^2,12^. Interestingly, a similar pattern was also noted in a Taiwanese population-based report by Lai et al.^13^ in which 41% of patients had adult-onset Coats’ disease. Similar to previous studies, we also found that childhood-onset cases more frequently presented at a more severe disease stage (Stage 3b) and were more likely to demonstrate diffuse exudation involving all quadrants and greater clock hours of retinal telangiectasia. Visual outcomes were also shown to be more favourable in the adult-onset group, a finding that supports another study by Shields et al.^14^ that demonstrated parallels between visual acuity outcomes and disease staging.

Recent management trends may be resulting in improved long-term outcomes for patients with Coats’ disease. Ong et al.^15^ compared patient outcomes over two decades and found that a combination of factors, including increased disease detection and more aggressive initial treatment strategies, have led to better final visual outcomes over time. Similarly, we found that long-term treatment outcomes were also comparatively better than those reported previously, especially in terms of globe salvage rate (only 2 eyes were enucleated, < 3%), which may be related to the earlier disease detection and subsequently lower overall disease severity at presentation in this population.

An evolution in treatment strategies over this timeframe, from cryotherapy to laser ablation to adjunctive anti-VEGF therapy, may be a key determinant of this improvement in patient outcomes. Our study supports the advantages of using laser therapy over cryotherapy as a primary treatment modality, as the latter was associated with a need for secondary interventions, which might be explained by its destructive action on retinal tissues and its potential to induce proliferative vitreoretinopathy.

Adjuvant anti-VEGF therapy is becoming increasingly more common in management of Coats’ disease^16–19^. Elevated VEGF levels in ocular fluids of patients with Coats’ disease has previously been reported, most likely secondarily due to retinal ischemia associated with the abnormal vasculature in this disease^20–22^. Furthermore, recent studies have demonstrated chorioretinal anastomoses and type 3 choroidal neovascularization in Coats’ disease patients^23^. Intravitreal anti-VEGF agents have safely been used in young patients to treat a wide variety of chorioretinal diseases over long periods of follow-up^24^, with no definite systemic or neurodevelopmental comorbidities identified, even in premature infants^25^. In fact, a study by Feng et al. suggested that anti-VEGF therapy may actually have higher efficacy in younger patients^26^, since paediatric Coats’ disease patients were shown to have higher aqueous concentrations of VEGF, interleukin (IL)-6 (a proinflammatory cytokine promoting increased vascular permeability), and IL-1ß (role in angiogenic diseases) than healthy controls. These findings also suggest that there may be an inflammatory component to this disease process, indicating that targeted corticosteroids in addition to anti-VEGF may have important roles to play in the management of Coats’ disease.

In our study, we observed that a greater number of anti-VEGF injections administered during the initial treatment period appeared to be associated with improved long-term visual outcomes. This finding occurred despite a lack of statistically quantifiable advantages in a subgroup analysis comparing patients who received only laser therapy with patients who received a combination of laser therapy and anti-VEGF injections. While multiple recent reports have demonstrated resolution of severe retinal detachments after anti-VEGF therapy^6^, a study by Ramasubramanian et al.^27^ cautioned against the use of anti-VEGF agents due to the development of vitreoretinal fibrosis and tractional detachments in eight patients. However, another more recent study by Daruich et al.^28^ showed that 40.6% of 69 patients had findings associated with extramacular fibrosis that were not associated with cryotherapy, laser therapy, or anti-VEGF treatment. Similarly, we did not identify any significant differences in the frequency of the development or progression of vitreoretinal fibrosis between eyes treated with laser therapy only and those treated with combination therapy (53% vs. 31%, *P* = 0.296).

Additionally, OCT angiography in patients with early Coats’ disease may offer clues to the mechanism by which anti-VEGF protects and improves central vision. In 13 patients, Schwartz et al.^29^ showed a significantly decreased macular vascular density and an enlarged foveal avascular zone in eyes with even Stage 2A Coats’ disease in comparison with the fellow eye, which preceded any clinical findings. These macular changes were similar to those that have been observed in patients with retinal vascular diseases caused by elevated VEGF levels resulting from hypoxic stress, such as diabetic retinopathy or retinal vein occlusions. Thus, treatment with anti-VEGF agents may reduce subclinical vascular leakage and may prevent accumulated damage to the macular area, leading to long-term visual improvement, no further compromise in retinal circulation, and prevention of macular ischemia^30^. Therefore, there may be a role for the use of anti-VEGF injection as an adjuvant therapy, and our results may provide further evidence for the long-term safety and efficacy of adjunctive anti-VEGF treatment in Coats’ disease.

Adjunctive corticosteroid treatment may also be a valuable tool for these patients, especially in those with adult-onset Coats’ disease and in those with severe exudation from diffuse leakage of telangiectatic capillaries^6^. Although corticosteroid treatment can potentially lead to cataract formation or elevated intraocular pressures^31^, dexamethasone intravitreal implants have been shown to be effective in the management of Coats’ disease^32,33^. Our study also supported this finding, as two cases with total retinal detachments from massive exudates showed dramatic decreases in size after dexamethasone implants.

Tractional retinal detachments may develop in response to laser ablation and/or cryotherapy, or from angiogenic dysregulation following anti-VEGF therapy^27,28^. The overall incidence rate of tractional retinal detachments over long-term follow-up detected in our study (19 eyes, 27%) is slightly higher than that reported previously, ranging from 10 to 21%^8,15^.

There were several limitations to this study. The study included an ethnically homogenous population, was retrospective in design, and did not include appropriate controls. Additionally, it was possible that patients with comparatively severe symptoms were referred more frequently to our tertiary centres, creating selection bias. The study’s strengths, however, included the relatively large cohort of patients with a rare disease who received long-term follow-up, allowing us to successfully identify several factors associated with final visual outcomes and with the need for secondary treatments. Future prospective studies are needed to determine the optimal treatment regimen for patients with Coats’ disease, which will likely involve a combination of anti-VEGF injections and laser ablative therapy.

In conclusion, we observed a higher proportion of adult-onset Coats’ disease in our patient population, which was associated with less severe disease staging at presentation and better visual outcomes. Using an aggressive first-line therapeutic approach involving combined laser therapy and anti-VEGF injections may be preferable to cryotherapy in these patients.

## Materials and methods

This retrospective study was conducted at two tertiary, referral-based, high-volume hospitals, Severance Eye Hospital and Gangnam Severance Hospital, both affiliated with Yonsei University College of Medicine. The study adhered to the tenets of the Declaration of Helsinki, and Gangnam Severance Hospital Institutional Review Board (IRB) approval was obtained (No. 3-2020-0139). The requirement for informed consent was waived by the Gangnam Severance Hospital IRB due to the retrospective nature of the study. Patients diagnosed with Coats’ disease who received follow-up between July 2000 and April 2020 were included.

For each included case, complete electronic medical records and multimodal imaging data were reviewed to confirm the diagnosis. Coats’ disease was diagnosed based on the presence of idiopathic retinal vascular telangiectasias and aneurysmal dilatations, with associated exudates and without findings indicative of other causes for exudation. Patients with inadequate data and/or partial records were excluded upon review. Patients were then grouped according to their age at presentation, with adult-onset Coats’ disease defined as those aged 18 years or above.

The following clinical characteristics and treatment outcomes were assessed for all included patients: (1) demographic data at presentation; (2) clinical data, including the stage of Coats’ disease, based on the classification scheme proposed by Shields et al.^7^ and vitreoretinal findings; (3) optical coherence tomography (OCT) findings, including the presence of cystoid macular oedema (CME) or epiretinal membrane (ERM); (4) first-line treatment modalities; (5) treatment failure due to disease recurrence, reactivation, and/or tractional retinal detachments requiring secondary interventions; and (6) long-term treatment outcomes, including development of complications and visual outcomes.

Treatment was determined by the retinal specialist, and although varied due to the specialist’s preference, treatment period, and disease severity, the overall treatment strategies could be categorized broadly into five regimens: (1) laser only, (2) laser combined with anti-VEGF injections, (3) cryotherapy, (4) surgical intervention (vitrectomy or scleral buckle), or (5) anti-VEGF only. Generally, patients with Stage 1 to 3a disease were treated using laser or cryotherapy, where leaking lesions were treated directly by laser (size 100–500 um, power of 150–200 mJ, depending on size and location) or by double freeze–thaw cryotherapy, with treatment applied to two or fewer quadrants per session to minimize side-effects^34^. Anti-VEGF injections (single dose or monthly doses up to 4 times) were given either alone or in combination with laser treatment^34,35^. Those with extensive retinal detachment determined to be not treatable using laser or cryotherapy, such as Stages 3b to 5 disease, underwent surgical intervention. Patients were grouped into these five broad, initial first-line treatment categories based on the stated treatment plan recorded on their respective electronic health records.

### Main outcome measures

Primary outcomes included the clinical characteristics of patients with Coats’ disease in Korea, including baseline demographic information, treatment strategies, and long-term outcomes, which were compared between groups based on age at disease presentation. In addition, these factors were compared to the current literature related to Coats’ disease, thus identifying potential clinically relevant differences in this specific population. Secondary outcomes included factors associated with final visual outcomes and factors associated with treatment failure (recurrence, reactivation, or tractional retinal detachment) or with a need for secondary interventions.

We performed statistical analyses using SPSS version 25.0 (IBM Corp., Armonk, NY, USA). The Kolmogorov–Smirnov test was used to analyse the sample distributions. The chi-squared and Student’s *t* tests were performed to compare data. Linear regression analysis was used to assess factors associated with final visual acuity, where the number of anti-VEGF injections performed indicated any injection whether in combination with laser or alone. Logistic regression analysis was used to assess the association of various clinical factors with eyes requiring secondary interventions. *P* < 0.05 were considered statistically significant.

## Data Availability

The datasets generated during and/or analysed during the current study are not publicly available due to privacy laws and policies in Korea, but are available from the corresponding author on reasonable request.
